# The Behavior of Matrix Metalloproteinases and Their Inhibitors in Colorectal Cancer

**DOI:** 10.3390/ijms131013240

**Published:** 2012-10-16

**Authors:** László Herszényi, István Hritz, Gábor Lakatos, Mária Zsófia Varga, Zsolt Tulassay

**Affiliations:** 1 Second Department of Medicine, Semmelweis University, H-1088 Budapest, Szentkirályi str. 46, H-1088, Hungary; E-Mails: istvan.hritz@gmail.com (I.H.); lakagab@yahoo.com (G.L.); varga.zsofia.maria@gmail.com (M.Z.V.); tulassay.zsolt@med.semmelweis-univ.hu (Z.T.); 2 First Department of Medicine, Fejér County Szent György Hospital, Székesfehérvár, H-8000, Hungary; 3 Department of Oncology, Szent László Hospital, Budapest, H-1097, Hungary

**Keywords:** matrix metalloproteinase, tissue inhibitor of matrix metalloproteinase, colorectal cancer, adenoma, tumor progression, invasion, metastasis, prognosis, biomarker

## Abstract

Matrix metalloproteinases (MMPs) play an important role in the degradation of extracellular matrix components crucial for tumor growth, invasion and metastasis. MMPs are controlled by natural inhibitors called tissue inhibitors of metalloproteinases (TIMPs). We and others have demonstrated that MMPs and TIMPs are especially important in the process of tumor invasion, progression and the metastasis of colorectal cancer (CRC). It has been proposed that MMPs and TIMPs might play a part not only in tumor invasion and initiation of metastasis but also in carcinogenesis from colorectal adenomas. Several recent studies demonstrated that high preoperative serum or plasma MMP-2, MMP-9 and TIMP-1 antigen levels are strong predictive factors for poor prognosis in patients with CRC and their determination might be useful for identification of patients with higher risk for cancer recurrence. MMP-9 and TIMP-1 have significant potential tumor marker impact in CRC. Their diagnostic sensitivity is consistently higher than those of conventional biomarkers. The pharmacological targeting of CRC by the development of a new generation of selective inhibitors of MMPs, that is highly specific for certain MMPs, is a promising and challenging area for the future.

## 1. Introduction

Colorectal cancer (CRC) is the fourth most common malignant neoplasm in men and the third in women worldwide. It accounts for an estimated 1.2 million new cancer cases and over 630,000 cancer death per year, almost 8% of all cancer deaths. Continents with a high incidence of CRC include North America and Europe. The lowest incidence is found in Africa, Asia and South America. In Japan and Eastern-Europe, the incidence of CRC has increased over recent years, probably due to a “Westernization” of lifestyle. CRC is the second most common newly diagnosed cancer and the second most common cause of cancer death in the European Union (EU) with an enormous health and economic burden, accounting for 1 out of 7 new cancers and 1 out of 8 cancer deaths: around 436,000 new cases and 212,000 deaths occur each year in Europe. Despite the significant advances in diagnosis, screening and treatment, the overall long-term outcome of curatively resected patients has not significantly changed in the last decades, the five-year survival rate being approximately 60%. More than a half of CRCs are still diagnosed only when the disease involves regional or distant structures. Different clinico-pathological prognostic factors have been revealed: tumor location, depth of tumor invasion, tumor differentiation (grade), tumor size and surgical procedure. The site of the tumor has been also investigated as a possible prognostic factor. Patients with rectal cancer have a poorer survival rate than those who suffer from colon cancer. The importance in predicting survival of tumor stage, as reflected in Dukes or TNM classification has been widely accepted. However, it would be very useful for clinicians to have additional preoperative prognostic indicators available, for instance to better identify patients who need adjuvant or neaoadjuvant treatment. Thus, there is a need for of additional tumor-related antigens at the time of clinical presentation, eligible as tumor markers and prognostic predictors in CRC [1–4].

## 2. Events during Tumor Invasion and Metastasis

Tumor invasion and metastasis is a multi-step event. It has been shown that cancer metastasis is a complex series of sequential processes: the initial transforming event; proliferation of transformed cells; the ability of tumor cells to avoid destruction by immune-response; nutrition supply to the tumor mass; local invasion and destruction of extracellular matrix components (ECM); migration of tumor cells; penetration of tumor cells through the blood vessel wall; embolization of tumor cells and adhesion to distant organs; arrest of cancer cells in the lumen of small blood vessels and lymphatics; reverse penetration of blood vessels and formation of distant metastases [5–10].

In the complex event of tumor invasion and metastasis, tumor cells are tightly interacted with basement membrane (BM) and ECM. Three steps have been suggested to describe the sequence of events during tumor cell invasion of ECM: attachment, matrix dissolution and migration. The first step is tumor cell attachment to the matrix. The attachment is mediated by tumor cell surface receptors, when tumor cells bind to the BM surface. This process is mediated by specific glycoproteins such as fibronectin and laminin. During the second step (local degradation of matrix by tumor cell-associated proteinases) tumor cells directly secrete enzymes to degrade ECM. Such proteinases can degrade both the structural collagenous proteins of the matrix and the attachment proteins. During the third step (migration), cancer cells are propelled across the BM and stroma through the zone of matrix proteolysis. Chemotactic factors can influence the direction of migration. Invasion of ECM is accomplished by cyclic reverberation of these three steps [7,10–15].

## 3. The Link between Inflammation and Cancer

Chronic inflammation is an indispensable risk factor in carcinogenesis. At least 20% of all cancers arise in association with chronic inflammation and infection [10,16–19].

Briefly, two pathways connect inflammation and cancer: (i) the intrinsic pathway is activated by genetic events (including chromosomal amplification, activation of oncogenes and inactivation of tumor-suppressor genes); (ii) in the extrinsic pathway, inflammatory circumstances promote cancer development. The coalescence of the intrinsic and extrinsic pathways activates transcription factors and finally a tumor-associated inflammatory milieu is created [10,20,21]. Cancer-associated inflammation is marked by the presence of specific inflammatory cells and inflammatory mediators, including cytokines and chemokines. They have an important role in tumor development and progression and are fundamental for keeping inflammation, promoting tumor growth and inhibiting immune-mediated tumor surveillance. Many of the above mentioned events are triggered by pro-inflammatory cytokines, such as interleukin-6 (IL-6) or tumor necrosis factor (TNF). TNF-α is fundamental in the initiation of chronic inflammation, activates nuclear factor-κB pathways, which plays an essential role in innate and adaptive immune responses, cell proliferation, apoptosis and carcinogenesis [10,16,22–25].

## 4. The Role of Proteolytic Enzymes

Proteolytic enzymes are major players in the breakdown and reconstitution of ECM in a variety of physiological and pathological processes, such as tissue remodeling, wound repair, inflammatory responses, angiogenesis, destructive diseases, as well as tumor invasion and metastasis. Proteinases are involved in physiological destructive and tissue remodeling processes including mammary gland involution, prostate involution, spermatogenesis, ovulation, throphoblast implantation, embryonal morphogenesis and neurite overgrowth. Activation and release of proteolytic enzymes has also been related to a variety of non-neoplastic pathological conditions, some of which involve acute as well as chronic inflammation and/or tissue degradation, such as degenerative diseases, osteoarthritis, pulmonary emphysema, chronic obstructive pulmonary disease, asthma, atherosclerosis, cardiovascular diseases, periodontitis or several other infectious disorders. Proteolytic enzymes are generally expressed in very low amounts and their transcription is regulated either positively or negatively by cytokines and growth factors such as inflammatory interleukins (IL-6, TNF) or transforming growth factors. Inflammatory cytokines enhance the dysfunction of proteinases, whereas proteolytic enzymes increase inflammation in the tissue. Proteinases selectively degrade various components of the ECM and release growth factors and cytokines that reside in the ECM. They also activate various latent growth factors, cytokines and chemokines and cleave cell surface proteins (cytokine receptors, cell adhesion molecules) [7,10,26].

There are several evidences implicating proteolytic enzymes in carcinogenesis and tumor development: (1) proteolytic enzymes are involved in normal tissue destruction which partially resemble cancer invasion and metastasis; (2) serum or tissue levels of proteolytic enzymes correlate with metastatic potential in experimental model systems; (3) the proteinase receptor (such as urokinase-type plasminogen activator receptor, UPA-R) blockade can inhibit metastatic spread; (4) inhibitors of proteinases or antibodies against certain proteolytic enzymes can prevent tumor invasion and metastasis in experimental conditions; (5) proteolytic enzymes are strong prognostic factor in many types of tumors [7,10,26,27].

The four categories of proteolytic enzymes (cysteine-, serine-, aspartic-, and metalloproteinases) are named and classified according to the essential catalytic component (usually an amino acid) in their active site [28].

We have previously shown that proteolytic enzymes such as cysteine and serine proteinases, may have a role not only in the process of gastrointestinal (GI) cancer invasion, or in the progression of GI precancerous lesions into cancer, but also they are widely distributed in GI tissues and they have been involved in processes of GI inflammation, tissue remodeling, angiogenesis and wound healing [29–33].

## 5. Matrix Metalloproteinases

Matrix metalloproteinases (MMPs) (a large family of Zn^2+^-dependent endopeptidases) play an important part in the degradation of ECM components, crucial for tumor growth, invasion and metastasis. To date 26 MMPs have been identified. MMPs are classified as gelatinases, collagenases, membrane-type, stromelysins and matrilysins, based mainly on the *in vivo* substrate specificity and sequence homology of the individual MMP.

Tumor cells produce proteolytic enzymes that can destroy the matrix barriers ambient the tumor, permitting invasion into surrounding connective tissues. MMPs are able to degrade virtually all components of the ECM and connective tissue surrounding the tumor cells and the basement membrane. It was initially believed that MMPs are being produced and secreted by tumor cells, degrading basement membrane and ECM components.

Now, we learned that MMPs are also produced by surrounding stromal cells, including fibroblasts and infiltrating inflammatory cells. It was initially believed that MMPs, via breakdown of the physical barrier, were primarily involved in tumor invasion. There is growing evidence, however, that MMPs have an expanded role, as they are important for the creation and maintenance of a microenvironment that facilitates growth and angiogenesis of tumors at primary and metastatic sites. In cancer, MMPs are involved in angiogenesis by regulating the bioavailability of vascular endothelial growth factor (VEGF) (e.g., MMP-9) and the cleavage of matrix-bound VEGF (MMPs-3, -7, -9). Further, MMPs can influence the balance between growth signals and growth-inhibiting signals (by activation of the epidermal growth factor (EGF) receptor and modulation of the transforming growth factor-β (TGF-β) pathway), regulate the induction of apoptosis by cleavage of Fas ligand (MMP-7), control inflammation (MMP-2, -3, -7, -8, -9, -12), play a part in the creation of metastatic niche (MMP-3, -9, -10) and invasive processes (MMP-1, -2, -7, -9, -13, -14) [34–41].

We and others have demonstrated that MMPs, among them particularly type IV collagenase MMP-9 (gelatinase B), are especially important in the process of tumor invasion and metastasis, but also in the remodeling and inflammatory processes in IBD [42–50]. Several MMPs are expressed by tumor cells in oral squamous cell carcinoma [51,52], prostate cancer [53], breast cancer [54], ovarian cancer [55–57] and in many GI tumors such as esophageal squamous cell and adenocarcinomas [58–60], gastric cancer [61,62], pancreatic cancer [63,64] or hepatocellular carcinoma [65,66]. In the specific case of MMP-9, we investigated the role of MMP-9 expression using immunohistochemical analysis in the development and progression of reflux esophagitis- Barrett’s esophagus-dysplasia-adenocarcinoma sequence in the esophagus. Increased immunohistochemical expression of MMP-9 in Barrett’s metaplasia-dysplasia-adenocarcinoma sequence as compared to normal tissue suggested its association with esophageal tumorigenesis. Increased expression of MMP-9 in Barrett with dysplasia compared to non-dysplastic metaplasia indicated that this alteration might be early event in carcinogenesis. We suggested that quantification of MMP-9 in Barrett’s esophagus might be useful to identify patients at higher risk for progression to esophageal adenocarcinoma [42].

In an immunohistochemical study we demonstrated that the mucosal up-regulation of MMP-9 correlated with the severity of inflammation in ulcerative colitis (UC), suggesting that the increased MMP-9 expression could contribute to the severity of mucosal damage in active UC. The gene expression microarray data and TaqMan real-time RT-PCR analysis confirmed our immunohistochemical results [49].

MMPs have been also considered as potential diagnostic and prognostic biomarkers in many types and stages of cancer [67,68].

## 6. Tissue Inhibitors of Matrix Metalloproteinases (TIMPs)

MMPs are controlled by endogenous tissue specific inhibitors called tissue inhibitors of metalloproteinases (TIMPs), which are secreted proteins. TIMPs bind and inhibit enzymatically active MMPs at a 1:1 molar stochiometric proportion thus inhibiting the proteolytic activity of MMPs. The impact of TIMPs is essential for the homeostasis of the ECM. The sensitive balance between MMPs and TIMPs is essential for many physiological processes in the gut [69–74]. We have recently demonstrated that not only MMPs but also TIMPs may contribute to the inflammatory and remodeling processes in IBD and serum TIMP-1 might be useful as additional biomarker in the assessment of IBD activity [50].

The imbalance between MMPs and TIMPs is an essential step in the development of GI malignancies and is of critical importance in early events of tumor progression. TIMPs might display a complex and dual influence on tumor progression and metastasis: on one hand they directly regulate and inhibit MMPs, on the other hand influence angiogenesis, inhibit the apoptosis of tumor cells, malignant transformation and facilitate tumor growth and metastasis [10,44,75–79].

Regulation of cell to cell and cell to matrix adhesion is controlled in normal cells, while disturbance in cell adhesion is common in human tumors. The relationship among cancer cells with the ECM and adjacent cells is accomplished through ECM components, adhesion molecules, proteinases and their endogenous tissue specific inhibitors. MMPs degrade the ECM and prepare the route for cancer cells to migrate, invade and spread to distant zone, forming metastasis. Active MMPs target ECM ligands for degradation (including laminin, collagens, vitronectin, fibronectin), resulting in ECM cancer cell detachment and remodeling. Active MMPs target also tumor cell adhesion receptors, such as integrins or E-cadherin and disrupt both cell- to cell and cell- to matrix adhesions. Tumor cell specific mechanisms, such as tumor angiogenesis or epithelial to mesenchimal transition (EMT) leads to increased MMPs activity. Ultimately, reduced MMPs inhibitory mechanisms may disrupt the MMPs/TIMPs ratio within the tumor microenvironment facilitating distant metastases [78–82].

Four TIMPs have been characterized in humans (TIMP-1, -2, -3 and TIMP-4), which share notable homology and structural identify at the protein level. TIMP-1 inhibits angiogenesis either directly by an as yet unknown mechanism or indirectly thorough restraining MMP-9-mediated release of vascular endothelial growth factor (VEGF) from matrix. TIMP-2 selectively block human microvascular endothelial cell growth *in vitro* in response to proangiogenic factors such as fibroblast growth factor-2 (FGF-2) or vascular endothelial growth factor A (VEGF-A). TIMP-2 could also suppress receptor tyrosine kinase signaling independent of MMP inhibition. TIMP-3 has been demonstrated to promote apoptosis in several *in vitro* systems. TIMP-3 has been also identified as a tumor suppressor for adherent malignant and normal cells. TIMP-3 inhibits adhesion of cells to ECM and promotes apoptosis through death-receptor-activated, caspase-8-mediated pathway. TIMP-4 enhances or inhibits the *in vivo* growth of tumor xenografts. It has also been shown that although TIMP-4 inhibits capillary endothelial cell migration *in vitro* it does not inhibit angiogenesis induced by FGF-2 in experimental systems [75,78,80–82].

Pathological angiogenesis is a hallmark of cancer. Angiogenesis is a complex process regulated by growth factors and by the force balance between endothelial cells traction stresses and ECM viscoelastic resistance. *In vitro* studies suggest that decreasing ECM stiffness can trigger an angiogenic switch. It has been demonstrated that MMPs should play an important role in this switching mechanism. Uncontrolled MMP activity results in tissue damage and functional alterations. MMP/TIMP physiological equilibrium is shifted in malignant tissues. During tumor progression there is an increase in secretion and activation of MMPs by either the tumor cells themselves or tumor-associated fibroblasts, launching the formation of tumor microenvironment. The increase of MMPs activities is initially inhibited by TIMPs. During tumor growth, more and more MMPs are secreted, overgrowing the local TIMPs secretion. This imbalance between MMPs and TIMPs can contribute to the ECM remodeling. Moreover, further tumor progression and growth drives local tissue hypoxia and MMP-mediated release of angiogenic factors. These steps facilitate the tumor-related angiogenic reaction, which also require MMP activity [78,79,83–86].

## 7. The Role of MMPs and TIMPs in Colorectal Cancer

### 7.1. Tissue Expression of MMPs and TIMPs in CRC

Several studies have demonstrated that the expression of several MMPs and TIMPs are enhanced in CRC. CRC is characterized by enhanced expression of several MMPs, such as MMP-1, MMP-2, MMP-7 or MMP-13, but one particular MMP, the type IV collagenase, MMP-9 (gelatinase B) is of special interest with respect to the development and progression of CRC. There is a multitude of published data by our group and others on MMP-9 and scarce data on other MMP members, hence the emphasis on MMP-9 in our review.

Previously some preliminary studies suggested that MMP-9 expression was related to prognosis [87–90]. More recently, in an immunohistochemical study we have demonstrated that tissue expression of MMP-9 was significantly higher in moderately (G2) and poorly (G3) differentiated tumors than in well differentiated (G1) cancers, as well as in advanced Dukes stages compared with Dukes stage A. We have shown diffuse strong MMP-9 expression in both tumor and stromal cells. For confirmation of our immunohistochemical results MMP-9 TaqMan real-time RT-PCR analysis was performed. Our RT-PCR results (using whole tissue lysates) correlate with the immunohistochemical behavior of MMP-9 in the colonic mucosa, showing a significantly higher expression of MMP-9 in cancer tissue compared to normal colonic mucosa [43].

Furthermore, recent immunohistochemical studies have confirmed that tissue MMP-9 can serve as an independent prognostic marker in CRC and quantification of MMP-9 expression may be valuable in finding patients who are at high risk of developing disease recurrence [91,92]. In a very recent study Chu *et al.* [93] demonstrated MMP-9 immunohistochemical expression was significantly positively correlated with depth of CRC invasion, lymph node metastasis and distant metastasis. Kaplan-Meier analysis of survival curves showed significantly worse survival for patients with positive MMP-9 staining than for those with MMP-9 negative cancers. Moreover, multivariate analysis adjusted for age, gender, tumor location, differentiation status and stage proved that MMP-9 expression was an independent marker of worse disease-free and overall survival, concluding that MMP-9 could serve as a novel prognostic marker that is additive to the tumor-node-metastasis (TNM) staging system.

Very recently Langers *et al.* [94] showed that high protein expression of MMP-9 and MMP-2 in normal mucosa at a distance of 5–10 cm from the tumor is also associated with worse five-year survival, indicating that increased MMP-9 and MMP-2 protein expression in normal mucosa of CRC patients is prognostic for survival.

In addition, it has also been demonstrated that not only MMP-9 and MMP-2, but also tissue expressions of MMP-1 and TIMPs might be useful prognostic markers and predictors of liver metastasis [95–97]. Moreover, very recently Gonzáles *et al.* [98] demonstrated that expressions of MMP-11 by fibroblasts and MMP-13 by tumor cells were also associated with poor prognosis.

It has been postulated that colonic and rectal carcinomas may have different mechanisms of carcinogenesis. Further, patients with rectal cancer are considered to have a poorer survival than those who suffer from colon cancer [99,100]. Recently it has been reported that in a specific case of rectal cancer tissue expression of gelatinases (MMP-2 and-9) also had a possible prognostic significance [101,102].

It has been proposed that MMPs and TIMPs might play a part not only in tumor invasion and initiation of metastasis but also in CRC carcinogenesis from colorectal adenomas. The adenomatous polyps are neoplastic tumors with a potential to progress into invasive CRC. The expression of MMP-9 and TIMP-1 in the normal mucosa-adenoma-dysplasia-adenocarcinoma sequence of the colon was studied by few authors. Tomita *et al*. [103] demonstrated that immunostaining of MMP-2 and MMP-9, as well as TIMP-1 and TIMP-2 increased gradually from tubular to villous adenomas, while *in situ* carcinomas showed a definite positive expression, concluding that the behavior of MMP-2, MMP-9 and TIMPs coincides with a multistep process of colonic tumorigenesis.

Our group has also shown that protein expression of MMP-9 in CRC was significantly higher compared to adenomas and the normal mucosa. In addition, we demonstrated higher tissue expression of MMP-9 in adenomas with HGD compared to other adenomas or normal colonic mucosa. In adenoma samples, dysplastic epithelial cells showed moderate intensive cytoplasmic MMP-9 expression, with a clear-cut differentiation between dysplastic and non-dysplastic areas, indicating that the overexpression of MMP-9 may be an early event in colorectal carcinogenesis [43].

### 7.2. Tissue Activity of MMPs in CRC

Using quantitative zymography, it has been suggested that the measured gelatinase (MMP-2 and MMP-9) activity also strongly correlate with the stage and prognosis of CRC [104,105]. In addition, Liabakk *et al*. [104] showed that activity of MMP-9 was significantly higher in CRC than in adenomas or normal colonic adenomas. In another study, Gimeno-Garcia *et al*. [106] observed elevated tissue activity of MMP-9 in patients with colorectal adenomas in comparison with normal mucosa. In this study, among neoplastic polyps, pro-MMP-9 activity was significantly higher in advanced *versus* non-advanced adenomas and in those harboring high-grade dysplasia (HGD), suggesting that MMP-9 might be a marker for early colorectal carcinogenesis. In a recent study, Murname *et al*. [107] demonstrated that also active MMP-2 activity might allow discrimination of colonic mucosa, adenomas with and without HGD and CRC, concluding that the ability of active MMP-2 to distinguish adenomas with HGD from adenomas without HGD may be essential in predicting additional CRC risk for an individual patient.

### 7.3. Genetic Analysis (Single-Nucleotide Polymorphism) of MMPs and TIMPs in CRC

Single-Nucleotide Polymorphism (SNP) is the most common type of genetic variation. Only a small part of polymorphisms is functionally essential. Most of the functional SNPs are located in the promoter region of the genes and are expected to influence gene expression. The effect of MMP polymorphisms on breast, lung and colorectal cancer has been reviewed previously by Decock *et al*. [108], while Langers *et al*. [109] summarized SNPs of MMPs and their inhibitors in gastrointestinal cancers, including CRC.

The most frequently studied MMP-9 polymorphism in CRC is the C to T substitution at position −1562 of the promoter region, which increases transcriptional activity. The association between the genotype and CRC susceptibility is ambiguous. In a population of 185 Korean CRC patients and 304 controls, the frequency of MMP-9 −1562 C homozygotes was significantly higher in CRC patients than in healthy controls [110]. In another Korean study the gene profiles of MMP-9 and TIMP-2 were assayed from 333 CRC patients. MMP-9 −1562 CC, TIMP-2 −418 GG and TIMP-2 303 GG genotypes were also more frequent in CRC patients than in controls [111]. None of the other studies demonstrated association between genotype and risk of CRC [112–115]. In addition, two important meta-analyses showed no significant association between the −1562 C/T MMP-9 polymorphism and CRC [116,117]. Xing *et al*. [112] reported a decrease of lymph node metastases in 137 Chinese CRC patients with the CC genotype of the MMP-9 −1562 SNP. Furthermore, they suggested that MMP-9 279 R allele may lead to a younger age of onset of CRC. The other studies did not find association with lymph node metastases, depth of infiltration, age and, any other clinicopathological variables or survival. Because of the heterogeneity of previous studies that have included a relatively small number of patients, further research on large cohorts of CRC patients and healthy controls is needed before a more conclusive judgment can be drawn from the influence of SNPs on CRC risk and prognosis.

### 7.4. Serum and Plasma MMPs and TIMPs in CRC

Several studies have compared the clinical significance of serum-plasma MMPs with TIMPs in the diagnosis of CRC, differentiation between CRC and colorectal adenomas, as well as their behavior in relation to clinicopathological parameters of CRC and to classical tumor markers.

### 7.5. Diagnostic Value of MMPs and TIMPs

The potential tumor marker impact of MMP-s and TIMPs has been extensively studied. It was clearly shown by several authors that MMP-9 and TIMP-1 have significant potential as biomarkers in CRC. Diagnostic sensitivity of MMP-9 and TIMP-1 was consistently higher compared with those of conventional biomarkers (CEA or CA 19–9). Is has been suggested that MMP-9 and TIMP-1 estimation likely have the greatest predictive impact when screened as part of a biomarker panel [118–120]. In a very recent study, Wilson *et al.* [121] evaluated the accuracy of MMP-9 for CRC in an asymptomatic population. From 748 patients overall, 46 cases of CRC were identified. Univariate analysis showed that increased serum MMP-9 concentration, demographic characteristics and behavioral factors were all significantly associated with presence of CRC. The final logistic regression model had a sensitivity and specificity of 79% and 70%, respectively.

In a population based Danish study 4509 symptomatic individuals referred to colonoscopy were prospectively included. Plasma was obtained before endoscopy and TIMP-1 and CEA levels were determined after the inclusion of all individuals. Colon and rectal cancers were detected in 184 and 110 individuals, respectively. A multivariate analysis showed that TIMP-1 and CEA were significant and independent markers of the presence of both colon and rectal cancers. This prospective validation study suggests the use of the combination of plasma TIMP-1 and CEA protein determinations as an additional support in early detection of CRC [122].

Although the fecal occult blood test (FOBT)-based colorectal screening is likely reducing the incidence and mortality of CRC, the test gives high false positive as well as negative results, therefore there is a need to improve the screening test, ideally to increase the positive predictive value. In a pilot study of 300 patients attending the Queen Elizabeth Hospital, high serum MMP-9 levels accurately predicted CRC in 77.3% of cases (sensitivity 77.9%, specificity 77.1%, positive predictive value [PPV] 44.6%, and negative predictive value [NPV] 95.8%). The results of this pilot study suggest that MMP-9 may be an effective secondary screening test [123]. It has been proposed by the same group that the use of MMP-9 as an adjunct to FOBT in a CRC screening program can improve the accuracy of screening and reduce the number of false positive tests [124].

### 7.6. Prognostic Value of MMPs and TIMPs

In a preliminary study we have demonstrated that serum antigen concentrations of MMP-2, MMP-7, MMP-9 as well as TIMP-1 and TIMP-2 were significantly higher in patients with CRC than in healthy controls. In addition, all examined parameters were significantly higher in patients with CRC than in patients with adenomas. Higher serum antigen concentrations of MMPs and TIMPs significantly correlated with tumor stage, nodal involvement and the presence of distant metastases. Our results from blood samples confirm previous results of tissue expressions concluding that MMPs and TIMPs play an important role in CRC invasion and metastasis, and they are also activated in premalignant colorectal adenomas. The increasing serum antigen concentrations of MMP-s and TIMPs coincide with a multistep process of colonic carcinogenesis. Furthermore, we suggested that measurement of MMPs and TIMPs might be useful in the assessment of preoperative tumor stage [125]. Our results are in agreement to the findings of Mroczko *et al*. [118] who revealed that serum concentrations of MMP-9 and TIMP-1 were significantly higher in adenoma patients compared with control group but lower than in patients with CRC.

Several studies confirmed that high preoperative serum or plasma MMP-2, MMP-9 and mainly TIMP-1 antigen levels are strong prognostic factors for patients with CRC and their determination might be useful for identification of patients with higher risk for cancer recurrence. Preoperative blood-levels of TIMP-1 were independent predictors of disease-free survival in patients with primary resectable CRC [118,119,126,127].

Very recently, Min *et al.* [128] showed that high serum levels of TIMP-1 were correlated with CRC liver metastasis and were significant predictive factors for poor prognosis following resection of synchronous liver metastasis.

In a pilot study, Pasternak *et al.* [129] measured MMPs in postoperative intraperitoneal fluid after rectal cancer surgery. They found that elevated MMP-8 and MMP-9 levels were markers for later development of anastomotic leakage after surgery. They suggest that MMPs appear to have an important role in the development of anastomotic leakage and may be promising pharmacological targets to protect anastomotic integrity.

The immunoassay kits of MMPs and TIMPs are usually designed for determination of concentrations in cell culture supernates, serum and/or plasma. For quantitative comparison in humans, plasma and/or serum concentrations are accepted to use, however the use of serum MMPs and TIMPs have been previously criticized due to its increased level compared with plasma estimation [130,131]. It is well known that MMPs are stored in macrophage and neutrophil granules, while most of TIMPs are secreted by platelets. Therefore, when using serum levels of MMPs and TIMPs, there are 3- to 5 fold higher levels than in corresponding EDTA or citrate plasma samples. Despite citrate-plasma being the suggested sample of choice for estimating circulating MMPs or TIMPS [132,133], serum sampling may still be useful provided that methods of collection and processing were standardized [121,134]. Thus, one should be aware of the pre-analytical pitfalls to avoid misinterpretation of data when determining MMP and/or TIMP levels. Further, when collecting samples it is recommended after centrifugation to aliquot and store samples at −20 °C or −80 °C or assay immediately. The time elapsed between blood sampling and centrifugation is associated with higher serum MMPs levels, with a suggested seven-fold increase after 2 h [135]. MMPs degrades during storage, even at −80 °C, therefore the repeated freeze-thaw cycles should be avoided, while TIMPs are stable and can be frozen/thawed for several times.

## 8. Predictive Value of MMPs and TIMPs in Response to Chemotherapy

It has also been suggested that TIMPs can predict individual responses to chemotherapy. In the study of Sörensen *et al.* [136] ninety patients with metastatic CRC were included. Plasma TIMP-1 and serum CEA were measured in samples obtained before the first cycle of first-line combination chemotherapy. It was shown that plasma TIMP-1 concentrations obtained before the first cycle of chemotherapy were significantly and independently associated with objective response, time to progression (TTP) and overall survival (OS) of patients with metastatic CRC receiving combination of irinotecan, 5-fluoruracil (5-FU), and folinic acid chemotherapy. CEA was not significantly associated with TTP or OS when TIMP-1 was included in the multivariable analysis. One explanation for these associations is that TIMP-1 protects cancer cells against the apoptotic stimuli that consecutively affect the cells. It has been demonstrated previously that TIMP-1 possesses antiapoptotic effects, which might be enhanced by administered chemotherapy. The antiapoptotic effect of TIMP-1 can be regulated in a MMP-dependent, and a MMP-independent way as a consequence of both direct effects on tumor cells and modulation of the tumor microenvironment [137]. *In vitro* and *in vivo* studies have shown that apoptotic effector molecules, such as caspases, are induced by degradation of ECM by MMPs, leading to apoptotic cell death. The anti-MMP function of TIMP-1 would indirectly inhibit apoptosis [137–139]. Knowing that TIMP-1 can induce chemoresistance in cancer cells *in vitro*, one can speculate whether TIMP-1 could be a real target for increasing tumor cell sensitivity to chemotherapy. Further prospective studies are needed to validate plasma TIMP-1 measurements in the prediction of response to chemotherapy.

Another study evaluated the effect of chemotherapy on plasma TIMP-1 antigen concentrations in comparison with CEA levels in patients with stage III CRC. Thirty patients with primary CRC, who had been intended curatively resected for stage III disease and scheduled for adjuvant chemotherapy, were prospectively included before the initiation of chemotherapy. Patients received 10–12 cycles of chemotherapy of a modified FOLFOX 6 regimen. Plasma CEA levels were stable during the first and second cycle of chemotherapy, while the plasma levels of TIMP-1 were directly affected by chemotherapy represented by a significant, but transient increase after two weeks following the second treatment and a recovery to normal three months later. According to this, TIMP-1 can be considered as an additional tool for monitoring chemotherapy in CRC [140]. The mechanism behind the observed increase in plasma TIMP-1 may be partly associated to cellular disintegration (tumor cells and/or blood cells) with subsequent release of soluble TIMP-1. The disintegration of platelets induced by chemotherapy and in particular to oxaliplatin is well known and might contribute to the raise of plasma TIMP-1 levels. Another explanation could be the up-regulation of TIMP-1 synthesis and release as a response to apoptosis induced by the chemotherapy [140–144].

Ramer *et al*. [145] demonstrated an essential role of TIMP-1 in the anti-invasive action of cisplatin on human cancer cells. It is suggested that this mechanism can play an important role in the antineoplastic actions of this prominent chemotherapeutic agent. In another study, the same group demonstrated that in human cancer cell lines increased expression of TIMP-1 mediates an anti-invasive effect of cannabinoids. It is well known, that cannabinoids, in addition to having palliative benefits in cancer therapy, have also been associated with anticarcinogenic effects [146]. Consistent with this finding, the anti-invasive action of several anticarcinogenic substances has been associated with elevated TIMP-1 levels [147–149].

Watanabe *et al*. [150] in a patient population comprising 25 patients with metastatic CRC treated with bevacizumab with either modified FOLFOX 6 or FOLFIRI, from whom tumor samples were available for gene expression analysis, showed that a model of genes for VEGF-A, thymidylate synthase and TIMP-3 predicted clinical response to bevacizumab therapy with an accuracy of 96%, sensitivity of 91%, specificity of 100%, and positive and negative predictive values of 100% and 93%, respectively, suggesting that the above predictive model may be useful in selection of CRC patients who would benefit from bevacizumab treatment.

## 9. Pharmacological Targeting of MMPs

Several therapeutic MMP inhibitors (MMPIs) have been developed to target MMPs, attempting to control their synthesis, secretion, activation and proteolytic activity. Several generations of synthetic MMPIs were under investigation in phase III clinical trials in recent years. Although there have been important preclinical and clinical studies on the agents targeting MMPs, most of these agents failed in clinical trials due to inefficacy and adverse side effect, thus they are yet not available for routine use as therapeutic agents [151–156].

The failure of MMPIs can be attributed to the following: (a) poorly defined predictive preclinical animal models for safety and efficacy; (b) limited knowledge of the variety of biological functions of MMPs; (c) poor selectivity of the MMPIs: due to homology between catalytic domains of MMPs none of the agents were highly selective for specific MMPs; (d) poor target validation for the targeted therapy: entry criteria excluded patients with early stage cancer, while MMPIs appear to be more active in early, rather than in late cancer stage (e) unexpected long-term drug intolerance reduced treatment compliance; (f) drug-dosage on short-term studies in healthy volunteers were not predictive of long-term therapeutic drug concentrations reached in cancer patients; (g) some MMPs exhibit antitumor activity; (h) MMPs are mainly produced by stromal cells, rather than the tumor cells themselves, therefore the exact cellular target of MMPIs was not precisely defined [157–159].

The most frequent severe adverse side effect associated with the clinical trials of MMPs was a musculoskeletal syndrome (MSS) that manifested as immobility and pain in the shoulder joints, arthralgias, contracture in the hands, and an overall impaired quality of life for patients. It was shown that development of MSS was time and dose-related. It has been suggested that development of MSS was the best indicator of dose optimization and successful MMP inhibition [160–162].

On the other hand, it should be always kept in mind that MMPs and TIMPs participate in regulation of tissue remodeling in healthy persons and in normal, non-cancerous tissue even in cancer patients. As such inhibition or blockade of these proteins will have influence on normal functions that may take place even in cancer patients. In addition, most CRC patients may also have concomitant disorders such as cardiovascular, hepatic or endocrine disorders, which also might be influenced by treatment with MMPIs [153,163,164].

The failure of MMPIs as cancer drugs in clinical practice suggests that our understanding of the molecular and cellular events involved in tissue remodeling is incomplete. In the light of our knowledge the proteolytic enzyme inhibitor approach seems no longer be sufficient because it does not affect the interactions of MMPs with cell surface proteins and consequents signaling [78,165].

The development of a new generation of selective inhibitors or monoclonal antibodies highly specific for certain MMPs is a promising area of research. New therapeutic strategies are focusing on more selective MMPIs: newly suggested inhibitors include peptides that block exosite-mediated cell surface interplay and/or function-blocking anti-MMP antibodies [78,166–171].

Furthermore, taken into consideration the high molecular complexity of tumor progression, combination of MMPIs with conventional chemotherapeutic or molecular targeted agents may also increase the effectiveness of oncological therapy [153,172].
